# Supplementary material for: Dynamic full-field optical coherence tomography module adapted to commercial microscopes allows longitudinal in vitro cell culture study
Source: Commun Biol. 2023 Sep 28;6:992. doi: 10.1038/s42003-023-05378-w (PMC10539404; doi:10.1038/s42003-023-05378-w)
Supplement: Supplementary file 2 — Supplementary material pdf [file 42003_2023_5378_MOESM2_ESM.pdf]

Supplementary note 1

To facilitate the understanding of 3D cell organisation in retinal organoids, as well as to account for the various cell organizations observed with D-FFOCT, a topology is introduced and explained schematically in this Supplement 1. Retinal organoids replicate retinal cell organisation [1] in a spherical configuration. Although they may vary in structure [2], retinal organoids will preferentially exhibit a spherical shape and cells will exhibit a radial orientation (Supplementary fig.1c-d). In a 28 days old retinal organoid, we observed as expected that retinal progenitor cells (RPCs) display a relatively symmetric shape with a major axis (see Fig. 1a-e in comparison to Supplementary fig. 1a) also radial to the spherical shape of the retinal organoid.

The purpose of this supplement is to illustrate why cells from the same cell type may appear with various apparent shapes depending on the plane and direction of observation. In order to provide such an illustration, we provide a schematic representation of the retinal organoid mainly composed of two radially superimposed layers composed of two different cell types with asymmetric shapes (green and yellow cells as sketched in Supplementary fig. 1a-b). The main axis of both cell types is chosen along the radius of the sphere, as postulated for RPCs in a retinal organoid. Supplementary fig. 1d displays a cross-section of the schematic retinal organoid along the depth axis (z).

An *en face* image of this sphere, at an equatorial plane, is displayed in Supplementary fig. 1c where both cell types consistently display the same shape.

Supplementary fig. 1e is an *en face* image of this sphere at a plane towards the bottom of the organoid, which hence captures cells with many different orientations. It results in the visualization of many different cell shapes associated with their orientations rather than because of morphological differences, making it difficult to associate a shape to a cell type and therefore to properly characterize the organoid.

Because D-FFOCT is an *en face* microscopy technique, we use this idealistic representation to postulate that efficient imaging of retinal organoids with circular symmetry can be obtained by performing 2D equatorial imaging. If the equatorial plane is not accessible, for example if the organoid is too large, we assume that an entire volume will encompass all cell types and layers of interest. Although volumetric acquisition enables the capture of most structures in an organoid, it requires much longer acquisition times and a larger dataset size.

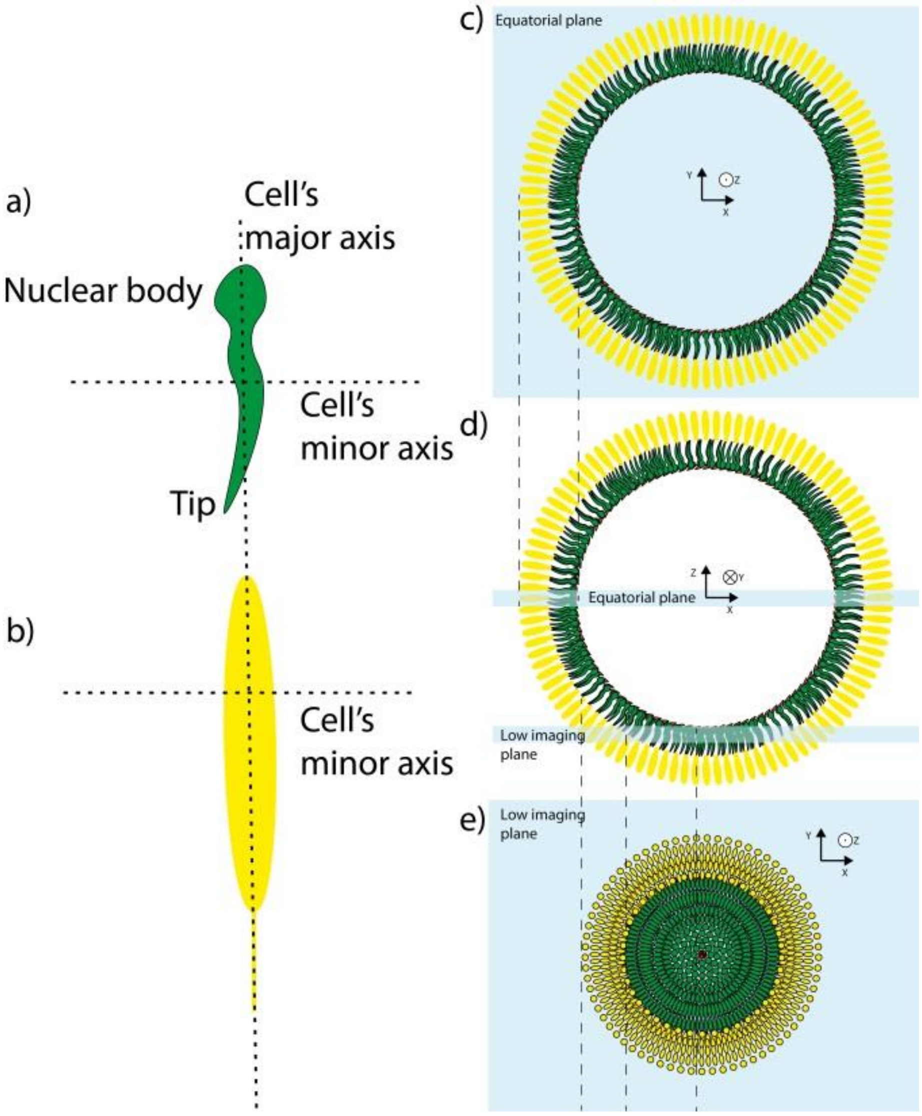

Supplementary fig. 1: retinal organoid schematic representation. Two spherical layers with different cell types are displayed at different cross sections in **c-e**. The two cell types are sketched in **a-b**.

<u>Supplementary note 2</u>

In Supplementary fig. 2, we display an *en face* view at a depth of 4 µm above the glass interface taken from the longitudinal volumetric acquisition displayed in Fig. 4. Axon-like structures, which likely correspond to retinal ganglion cell (RGC) axons [3, 4], are extending outwardly from the retinal organoid from day 29 (d29) onwards, thus significantly earlier than observations made at a depth of 50 µm displayed Fig. 4.

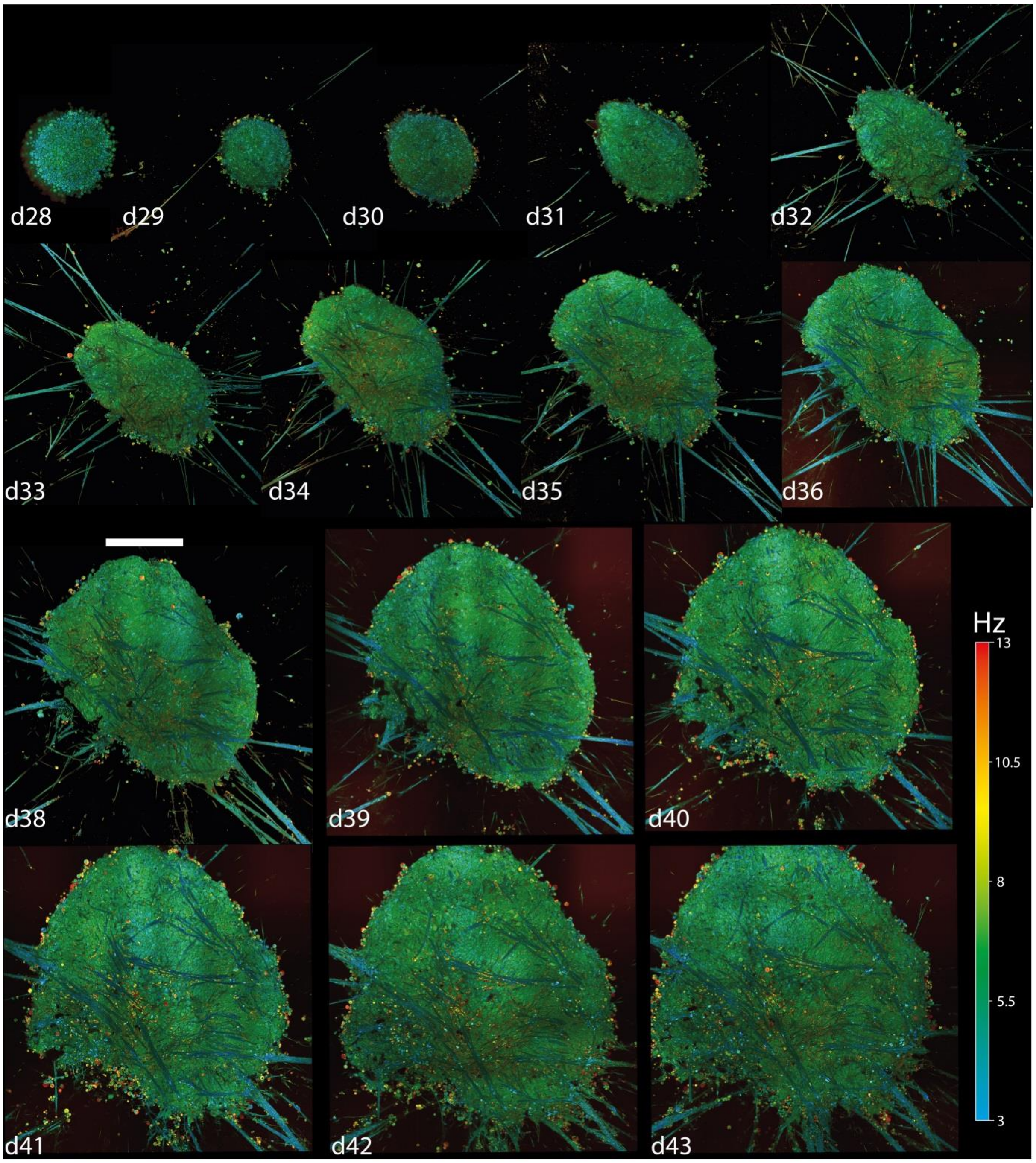

Supplementary fig. 2: D-FFOCT volumetric and longitudinal imaging of a single organoid at a depth of 4 µm, across 17 days. Hue scales from 3 to 13 Hz mean frequency. Image mosaicking covers 406x406 µm2, 2928x2928 pixels, (3x3), at day 27 (d27) to 717x717 µm2, 5163x5163 pixels, (6x6), at day 43 (d43). Scale bar, 50µm.

Supplementary note 3

On the organoid shown in Suppplementary fig. 2, at such low depth, we also observe retinal progenitor cells (RPCs) during mitosis, as shown in Supplementary fig. 3a,d (white arrows, and zoom-ins displayed in Supplementary fig. 3b,e). More strikingly at this plane as compared to the plane at 50 µm displayed in the main text, and because of the radial organization of the retinal organoid (as illustrated in Supplementary fig. 1), the RPC tips (Supplementary fig. 1a) localised at the edge of the retinal organoid show a smaller cross section than regular RPCs (3 µm instead of 8 µm on average) found in the middle of the same imaging plane, as well as a lower activity (3 Hz). We observed that some RPCs have detached from the surface of the retinal organoid and display a round red and yellow saturated profile (9-10.5 Hz) indicating cell stress [5].

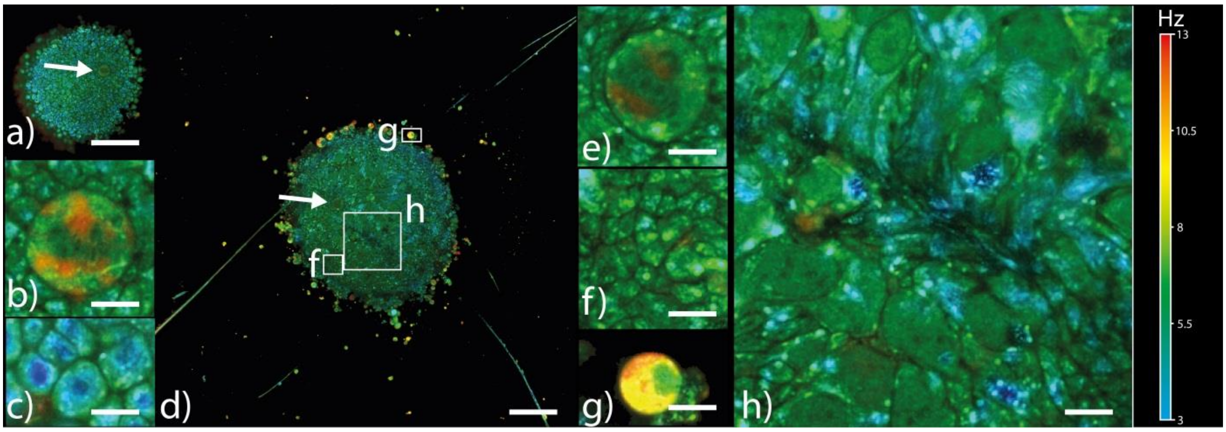

Supplementary fig. 3: Highlights on different structures of a retinal organoid at day 27 (d27 a-c) and 29 (d29 d-h). Hue scales from 3 to 13 Hz mean frequency. White arrows highlight mitotic states in retinal progenitor cells and highlights of these cells are shown in **b** and **e**. Scale bar is 50 µm for **a** and **d**, 5 µm for **b-c** and **e-g**, 6 µm in **h**.

Finally, we observe at this depth the beginning of a rosette formation (Suppplementary fig. 3d, highlighted in Suppplementary fig. 3h, from d29 (Suppplementary fig. 2). This event supports our observations on the genesis of rosette originating from external retinal organoid lumen.

Supplementary note 4

Additional Z-Stacks on retinal organoids between 160 and 275 days old were imaged using D-FFOCT. One example from this dataset, at d160, is displayed in Supplementary fig. 4, at 80 µm depth using a LED at 730 nm (M730L5, Thorlabs, Newport, NJ, USA), acquired at 100 Hz.

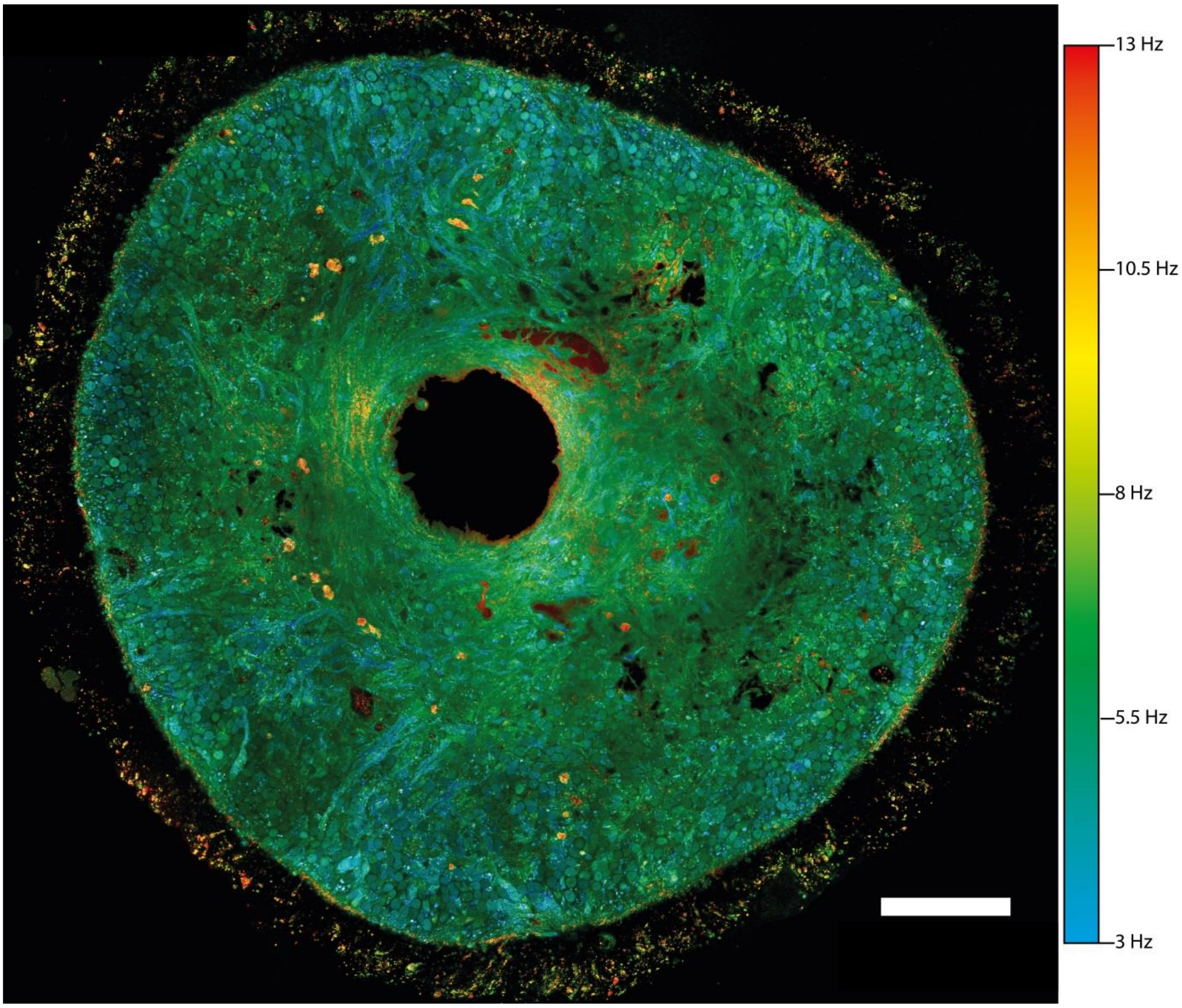

Supplementary fig.4: retinal organoid imaged using D-FFOCT. Hue scales from 3 to 13 Hz mean frequency. Image mosaicking covers 815x772 μm^2, 5868x5560 pixels, (7x7), at d160. Depth, 80µm. Scale bar, 100µm.

Supplementary note 5

In order to visualize how the acquisitions are carried out in this work, a schematic representation of the data workflow is displayed in Suppplementary fig. 5, as described in the methods section “Optimization of data workflow” in the manuscript text.

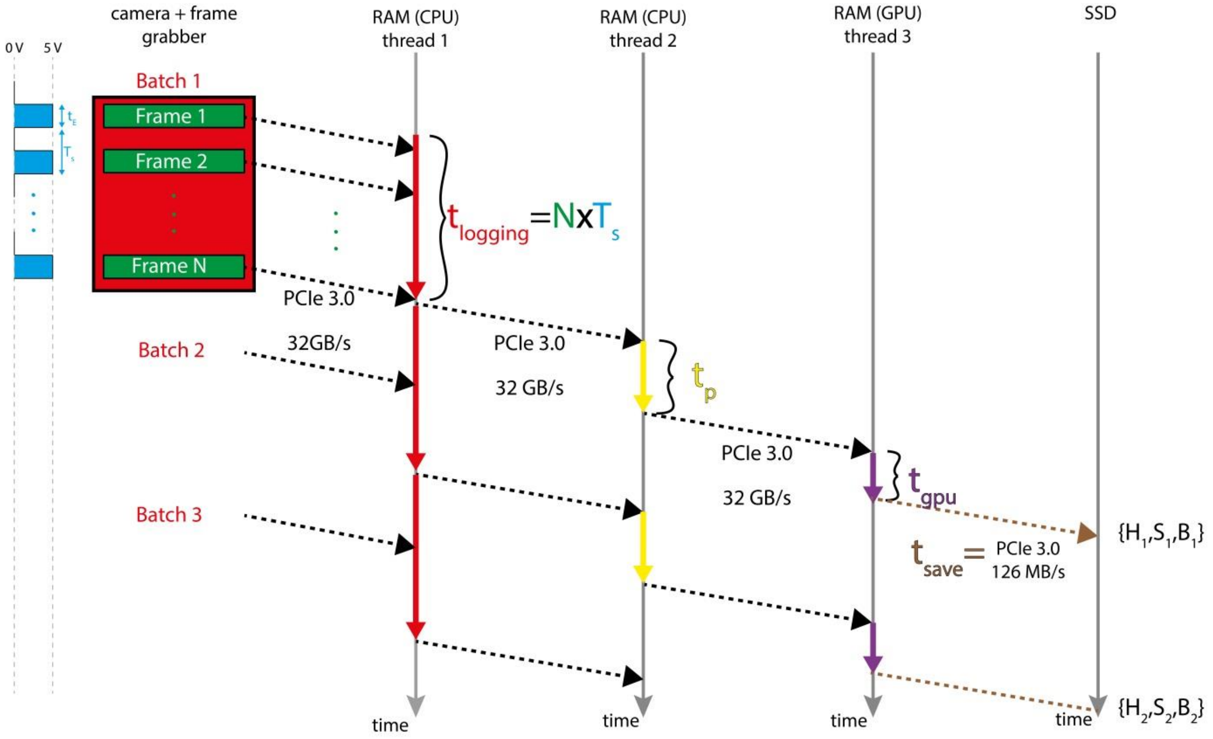

Supplementary fig. 5: Schematic of the data work flow used for generating D-FFOCT images. A transistor-transistor logic 0-5 V signal was generated from an acquisition card (NI cDAQ-9174, National Instruments,TX, USA) enabling synchronisation of the acquisition with the position of the piezoelectric for FFOCT 2-phases imaging. Period of time (t_E) at 5 V corresponding to the exposure time before capacitor discharge. As a result, images at 1/T_s speed were acquired. N frames were acquired per batches before being sent to a parallel thread on the computing process unit (CPU) (CORSAIR 4000D AIRFLOW, AMD Ryzen 5 3600 (3.6 GHz / 4.2 GHz), California, USA) random access memory (RAM) (8x Vengeance LPX 1X16GB 3000MHZ, Corsair, California, USA). Whilst a second batch is being acquired, the first batch, now transferred on the CPU RAM, used as a buffer, is transferred in MATLAB working space on a second CPU RAM thread. A third thread on a graphic unit process (GPU) is used for fast calculation in native flop data type, such as fast-Fourier transform. A fourth CPU RAM thread gathers the post-processed data and save them on a solid-state disk. t_p is the time to transfer the data onto the computing RAM, t_{gpu} the post-processing time on graphical processing unit (GPU) and t_{save} the time it takes to save the post-processed data.

Supplementary references

[1] A. Fatehullah, S. H. Tan, and N. Barker, “Organoids as an in vitro model of human development and disease,” *Nature Cell Biology*, vol. 18, no. 3, pp. 246–254, feb 2016.

[2] J. Friedrich, C. Seidel, R. Ebner, and L. A. Kunz-Schughart, “Spheroid-based drug screen: considerations and practical approach,” *Nature Protocols*, vol. 4, no. 3, pp. 309–324, feb 2009.

[3] C. M. Fligor, K. B. Langer, A. Sridhar, Y. Ren, P. K. Shields, M. C. Edler, S. K. Ohlemacher, V. M. Sluch, D. J. Zack, C. Zhang, D. M. Suter, and J. S. Meyer, “Three-dimensional retinal organoids facilitate the investigation of retinal ganglion cell development, organization and neurite outgrowth from human pluripotent stem cells,” *Scientific Reports*, vol. 8, no. 1, sep 2018.

[4] S. Reichman, A. Slembrouck, G. Gagliardi, A. Chaffiol, A. Terray, C. Nanteau, A. Potey, M. Belle, O. Rabesandratana, J. Duebel, G. Orieux, E. F. Nandrot, J.-A. Sahel, and O. Goureau, “Generation of storable retinal organoids and retinal pigmented epithelium from adherent human iPS cells in xeno-free and feeder-free conditions,” *Stem Cells*, vol. 35, no. 5, pp. 1176–1188, feb 2017.

[5] K. Groux, A. Verschueren, C. Nanteau, M. Clémençon, M. Fink, J.-A. Sahel, C. Boccara, M. Paques, S. Reichman, and K. Grieve, “Dynamic full-field optical coherence tomography allows live imaging of retinal pigment epithelium stress model,” *Communications Biology*, vol. 5, no. 1, jun 2022.

[6] J. Scholler, V. Mazlin, O. Thouvenin, K. Groux, P. Xiao, J.-A. Sahel, M. Fink, C. Boccara, and K. Grieve, “Probing dynamic processes in the eye at multiple spatial and temporal scales with multimodal full field OCT,” *Biomedical Optics Express*, vol. 10, no. 2, p. 731, jan 2019.

[7] J. Scholler, K. Groux, O. Goureau, J.-A. Sahel, M. Fink, S. Reichman, C. Boccara, and K. Grieve, “Dynamic full-field optical coherence tomography: 3d live-imaging of retinal organoids,” *Light: Science & Applications*, vol. 9, no. 1, aug 2020.
