# Supplementary material for: Dynamic full-field optical coherence tomography module adapted to commercial microscopes allows longitudinal in vitro cell culture study
Source: Commun Biol. 2023 Sep 28;6:992. doi: 10.1038/s42003-023-05378-w (PMC10539404; doi:10.1038/s42003-023-05378-w)
Supplement: Supplementary file 3 — Description of Supplementary Materials [file 42003_2023_5378_MOESM3_ESM.docx]

**Description of Additional Supplementary Files**

**File name:** Supplementary data

**Description:** TUNEL and CELLS data of graphs in Figures 6 and 7 in excel format.

**File name:** Supplementary movie

**Description:** Timelapse over 11 hours on a locked plane at 50µm depth, field size 406 µm x 406 µm, on the organoid from Fig. 4.
