# Supplementary material for: Dynamic full-field optical coherence tomography module adapted to commercial microscopes allows longitudinal in vitro cell culture study
Source: Commun Biol. 2023 Sep 28;6:992. doi: 10.1038/s42003-023-05378-w (PMC10539404; doi:10.1038/s42003-023-05378-w)
Supplement: Supplementary file 6 — Reporting summary checklist [file 42003_2023_5378_MOESM6_ESM.pdf]

Corresponding author(s): Kate Grieve
COMMSBIO-23-0629

Last updated by author(s): Jul 27, 2023

Reporting Summary

Nature Portfolio wishes to improve the reproducibility of the work that we publish. This form provides structure for consistency and transparency in reporting. For further information on Nature Portfolio policies, see our Editorial Policies and the Editorial Policy Checklist.

Statistics

For all statistical analyses, confirm that the following items are present in the figure legend, table legend, main text, or Methods section.

n/a	Confirmed	
☐	☒	The exact sample size (*n*) for each experimental group/condition, given as a discrete number and unit of measurement
☐	☒	A statement on whether measurements were taken from distinct samples or whether the same sample was measured repeatedly
☐	☒	The statistical test(s) used AND whether they are one- or two-sided<br>*Only common tests should be described solely by name; describe more complex techniques in the Methods section.*
☒	☐	A description of all covariates tested
☒	☐	A description of any assumptions or corrections, such as tests of normality and adjustment for multiple comparisons
☐	☒	A full description of the statistical parameters including central tendency (e.g. means) or other basic estimates (e.g. regression coefficient) AND variation (e.g. standard deviation) or associated estimates of uncertainty (e.g. confidence intervals)
☒	☐	For null hypothesis testing, the test statistic (e.g. *F*, *t*, *r*) with confidence intervals, effect sizes, degrees of freedom and *P* value noted<br>*Give P values as exact values whenever suitable.*
☒	☐	For Bayesian analysis, information on the choice of priors and Markov chain Monte Carlo settings
☒	☐	For hierarchical and complex designs, identification of the appropriate level for tests and full reporting of outcomes
☒	☐	Estimates of effect sizes (e.g. Cohen's *d*, Pearson's *r*), indicating how they were calculated

Our web collection on statistics for biologists contains articles on many of the points above.

Software and code

Policy information about availability of computer code

Data collection	The microscope functionalities as well as the 2D XY translation stage are interfaced through JAVA functions developed by µManager [86]—in which MATLAB is coded—and the other instruments are controlled through MATLAB (2021b). A dedicated graphic user interface (GUI) was created from scratch for this setup.<br>Mosaics were reconstructed from tiles with 50% overlapping using MIST (Microscopy Image Stitching Tool) [87], a plug-in available on Fiji 1.54b [88].
Data analysis	Thedata processing framework was accomplished with MATLAB (2021b), using the toolbox Imaqtool. The whole custom cell population analysis pipeline is implemented in MATLAB. The code can be provided by the authors upon reasonable request. Our custom-developed method was validated using a different workflow, and results were compared. Using the Trainable WEKA Segmentation [89] on Fiji 1.54b [90], two classes were found (i.e. cells versus background), generating a probability map that was then thresholded and binarized using the Otsu algorithm. The Analyze Particle tool was then used in order to count the cells.<br>Codes for acquisition and analysis can be made available upon request to authors.

For manuscripts utilizing custom algorithms or software that are central to the research but not yet described in published literature, software must be made available to editors and reviewers. We strongly encourage code deposition in a community repository (e.g. GitHub). See the Nature Portfolio guidelines for submitting code & software for further information.

Data

Policy information about availability of data

All manuscripts must include a data availability statement. This statement should provide the following information, where applicable:

- Accession codes, unique identifiers, or web links for publicly available datasets
- A description of any restrictions on data availability
- For clinical datasets or third party data, please ensure that the statement adheres to our policy

Data can be made available upon reasonable request from the authors.

Human research participants

Policy information about studies involving human research participants and Sex and Gender in Research.

Reporting on sex and gender	*Use the terms sex (biological attribute) and gender (shaped by social and cultural circumstances) carefully in order to avoid confusing both terms. Indicate if findings apply to only one sex or gender; describe whether sex and gender were considered in study design whether sex and/or gender was determined based on self-reporting or assigned and methods used. Provide in the source data disaggregated sex and gender data where this information has been collected, and consent has been obtained for sharing of individual-level data; provide overall numbers in this Reporting Summary. Please state if this information has not been collected. Report sex- and gender-based analyses where performed, justify reasons for lack of sex- and gender-based analysis.*
Population characteristics	*Describe the covariate-relevant population characteristics of the human research participants (e.g. age, genotypic information, past and current diagnosis and treatment categories). If you filled out the behavioural & social sciences study design questions and have nothing to add here, write "See above."*
Recruitment	*Describe how participants were recruited. Outline any potential self-selection bias or other biases that may be present and how these are likely to impact results.*
Ethics oversight	*Identify the organization(s) that approved the study protocol.*

Note that full information on the approval of the study protocol must also be provided in the manuscript.

Field-specific reporting

Please select the one below that is the best fit for your research. If you are not sure, read the appropriate sections before making your selection.

☒ Life sciences ☐ Behavioural & social sciences ☐ Ecological, evolutionary & environmental sciences

For a reference copy of the document with all sections, see nature.com/documents/nr-reporting-summary-flat.pdf

Life sciences study design

All studies must disclose on these points even when the disclosure is negative.

Sample size	All data were from biological triplicate samples
Data exclusions	No data were excluded from the analysis. Exclusion criteria were not pre-established
Replication	The reproductibility was verify using biological triplicate samples
Randomization	Biological samples collected at specific time were allocated into the experimental group associated without bias
Blinding	Blinding was not relevent in our study.

Reporting for specific materials, systems and methods

We require information from authors about some types of materials, experimental systems and methods used in many studies. Here, indicate whether each material, system or method listed is relevant to your study. If you are not sure if a list item applies to your research, read the appropriate section before selecting a response.

Materials & experimental systems

n/a	Involved in the study	
☐	☒	Antibodies
☐	☒	Eukaryotic cell lines
☒	☐	Palaeontology and archaeology
☒	☐	Animals and other organisms
☒	☐	Clinical data
☒	☐	Dual use research of concern

Methods

n/a	Involved in the study	
☒	☐	ChIP-seq
☒	☐	Flow cytometry
☒	☐	MRI-based neuroimaging

Antibodies

Antibodies used

Antigen Species Dilution Source
Primary antibodies
BRN3A Mouse monoclonal 1/250 Millipore (MAB1585)
PAX6 Rabbit polyclonal 1/2000 Millipore (AB2237)
VSX2 Mouse monoclonal 1/200 Santa Cruz (SC365519)
Secondary antibodies
Alexa fluor 488 anti-mouse Donkey 1/300 Jackson ImmunoResearch
715-545-150
Alexa fluor 488 anti-rabbit Donkey 1/300 Jackson ImmunoResearch
711-545-152
Alexa fluor 647 anti-mouse Donkey 1/300 Jackson ImmunoResearch

Validation

Relevant citations validating Antibodies:

- Reichman S, Terray A, Slembrouck A, Nanteau C, Orieux G, Habeler W, Nandrot EF, Sahel JA, Monville C, Goureau O. From confluent human iPS cells to self-forming neural retina and retinal pigmented epithelium. Proc Natl Acad Sci U S A. 2014 Jun 10;111(23):8518-23. doi: 10.1073/pnas.1324212111.

- Reichman S, Slembrouck A, Gagliardi G, Chaffiol A, Terray A, Nanteau C, Potey A, Belle M, Rabesandratana O, Duebel J, Orieux G, Nandrot E, Sahel JA and Goureau O. Generation of storable retinal organoids and retinal pigmented epithelium from adherent human iPS cells in xeno-free and feeder-free conditions. Stem Cells. 2017 May;35(5):1176-1188. doi: 10.1002/stem.2586.

- Slembrouck-Brec A, Nanteau C, Sahel JA, Goureau O, Reichman S. Defined Xeno-free and Feeder-free Culture Conditions for the Generation of Human iPSC-derived Retinal Cell Models. J Vis Exp. 2018 Sep 6;(139):57795. doi: 10.3791/57795.

Eukaryotic cell lines

Policy information about cell lines and Sex and Gender in Research

Cell line source(s)

hiPSC source:
-hiPSC-5F (hPSC REG: IDVi005) derived from human retinal Müller glial cells as previously described in Slembrouck-Brec et al., 2019.

Authentication

The hiPSC_5f line authenticated by the European cell bank number (hPSC REG) IDVi005.

Mycoplasma contamination

We confirm that all cell lines were tested negative for mycoplasma contamination.

Commonly misidentified lines
(See ICLAC register)

We did not use misidentified lines
